# Taxallnomy: an extension of NCBI Taxonomy that produces a hierarchically complete taxonomic tree

**DOI:** 10.1186/s12859-021-04304-3

**Published:** 2021-07-29

**Authors:** Tetsu Sakamoto, J. Miguel Ortega

**Affiliations:** 1 BioME - Bioinformatics Multidisciplinary Environment, Instituto Metrópole Digital (IMD), Universidade Federal Do Rio Grande Do Norte (UFRN), Natal, RN Brazil; 2 Laboratório de Biodados, Departamento de Bioquímica E Imunologia, Instituto de Ciências Biológicas (ICB), Universidade Federal de Minas Gerais (UFMG), Belo Horizonte, MG Brazil

**Keywords:** NCBI Taxonomy, Taxonomic rank, Taxonomic lineage, No rank, Linnaean system

## Abstract

**Background:**

NCBI Taxonomy is the main taxonomic source for several bioinformatics tools and databases since all organisms with sequence accessions deposited on INSDC are organized in its hierarchical structure. Despite the extensive use and application of this data source, an alternative representation of data as a table would facilitate the use of information for processing bioinformatics data. To do so, since some taxonomic-ranks are missing in some lineages, an algorithm might propose provisional names for all taxonomic-ranks.

**Results:**

To address this issue, we developed an algorithm that takes the tree structure from NCBI Taxonomy and generates a hierarchically complete taxonomic table, maintaining its compatibility with the original tree. The procedures performed by the algorithm consist of attempting to assign a taxonomic-rank to an existing clade or “no rank” node when possible, using its name as part of the created taxonomic-rank name (e.g. *Ord_*Ornithischia) or interpolating parent nodes when needed (e.g. *Cla_of_*Ornithischia), both examples given for the dinosaur *Brachylophosaurus* lineage. The new hierarchical structure was named Taxallnomy because it contains names for all taxonomic-ranks, and it contains 41 hierarchical levels corresponding to the 41 taxonomic-ranks currently found in the NCBI Taxonomy database. From Taxallnomy, users can obtain the complete taxonomic lineage with 41 nodes of all taxa available in the NCBI Taxonomy database, without any hazard to the original tree information. In this work, we demonstrate its applicability by embedding taxonomic information of a specified rank into a phylogenetic tree and by producing metagenomics profiles.

**Conclusion:**

Taxallnomy applies to any bioinformatics analyses that depend on the information from NCBI Taxonomy. Taxallnomy is updated periodically but with a distributed PERL script users can generate it locally using NCBI Taxonomy as input. All Taxallnomy resources are available at http://bioinfo.icb.ufmg.br/taxallnomy.

## Background

Any biological data are tightly linked to taxonomic data and several bioinformatics analyses depend on taxonomic information to achieve their objectives. Metagenomics, clinical forensic medicine, and other fields rely on fully-annotated taxonomic data to identify and group organisms present in a sample, often summarizing the results to a taxonomic-rank such as family, order, class, or phylum. Furthermore, any discussion made from evolutionary analyses refers to the taxonomic classification proposed so far. Taxonomic information can be obtained from several taxonomic databases, like the Catalogue of Life [1], which provides the taxonomic backbone to other projects such as Tree of Life [2], Encyclopedia of Life [3], and GBIF [4]. Information provided by those databases is supported by taxonomy experts that feed other databases that cover a more specific clade, like FishBase [5], AmphibiaWeb [6], AnimalBase [7], and others. However, any analyses that involve molecular sequences are dependent on the NCBI Taxonomy [8], a reference taxonomic database with a huge compilation of taxonomic names and lineages of organisms that have a register of their DNA or protein sequence in one of the databases comprising the International Nucleotide Sequence Database Collaboration (INSDC) [9]. Since INSDC comprises the three main molecular sequence repositories, GenBank, ENA, and DBJJ, the information provided by NCBI Taxonomy is broadly used in biological databases covering diverse subjects that rely on data from INSDC, such as UniProtKB [10], Ensembl [11], Pfam [12], SMART [13], Panther [14], OMA [15] and miRBase [16]. Moreover, other main primary biological databases, such as PDB [17], ArrayExpress [18], and KEGG [19] link their accessions to taxonomic data from the NCBI Taxonomy database, demonstrating the undeniable contribution of this database to several bioinformatics fields.

The taxonomic classification comprising the NCBI Taxonomy follows the phylogenetic taxonomy scheme with the topology reflecting a consensus of views from taxonomic and molecular systematic literature [8] and the information is organized in a tree. Each node of the tree represents a taxon, and each of them has a taxonomic name and a taxonomic identifier (txid) associated. Besides, some nodes may have a taxonomic-rank, which is similar to those used on the Linnaean classification system, such as Phylum, Class, Order, etc. which serve as important references of taxonomic classification for many analyses. Several bioinformatics approaches rely on the rank-based classification provided by NCBI Taxonomy to make, for instance, taxonomic profiles of metagenomic data or to assist the taxonomic classification of sequence data according to given ranks of their lineages. Besides the large use of rank information in the bioinformatics community, however, there are some important issues to be considered when managing these data. When querying for a group of organism lineages, we could observe that some of them are lacking some ranks. In a consultation on NCBI Taxonomy performed in November 2020, most of the cyanobacteria, such as *Microcystis aeruginosa* (NCBI:txid:1126), had no taxon with the Class rank. When our group has started developing Taxallnomy, *Arabidopsis* did not have Class and pig did not have Order ranks. However, if we look further in the taxonomic lineages, we find some taxa without taxonomic-rank, denoted as “no rank” or “clade” taxa, included in the tree to add phylogenetic information to the taxonomy base, pointing out monophyletic groups. Those might be useful nodes to be borrowed to represent preliminary added taxonomic-ranks. When that is not possible, the interpolation of new nodes without affecting the original hierarchy would be the solution.

These issues may be due to the uncertainty or conflict amongst experts on the classification of this group and turn to make hierarchical taxonomic-ranks of NCBI Taxonomy incomplete, or the experts are not missing some taxonomic-ranks in some lineages. Because of that, a simple query regarding the taxonomic-ranks, such as “How many distinct taxa of class rank are represented in this data?” could become a difficult task. For instance, if the class for *M. aeruginosa* and several non-assigned classes of cyanobacteria are present they will all be counted as “NULL” in a computational database such as MySQL, therefore grouping non-related counts. For such analyses, a hierarchically complete taxonomic tree incorporating “all” taxonomic-ranks could be of great benefit. Thus, in this work, we developed an algorithm that carefully takes the taxonomic tree provided by NCBI Taxonomy and generates a hierarchical taxonomic tree in which all lineages have the same depth and all hierarchical levels corresponding to a taxonomic-rank, thus it can be handled as a table of 41 columns. Additionally, the table can regenerate the original tree with all nodes if desired for the computational analyses. The final database was named Taxallnomy because it provides taxonomic names for all taxonomic-ranks in a lineage comprised in NCBI Taxonomy. Taxallnomy thus programmatically proposes *provisional* names for all gaps in taxonomic-rank in NCBI Taxonomy, favoring bioinformatics analysis and maybe inspiring curators on proposing the appropriated names for novel taxonomic-ranks. The procedure acts in such a way that does not harm the NCBI Taxonomy classification schema. Names of the proposed ranked taxa are generated by appending prefixes to existing node names, so they will not be mistaken as unappropriated novel taxa, which might be created after appropriated nomenclature by taxonomists. Taxallnomy is in a tab-delimited format, making it easy to access all members of a given clade (e.g. all species *Cla_of_*Testudines, where turtles are classified). Users can access and explore the hierarchical structure of the Taxallnomy database at its website at http://bioinfo.icb.ufmg.br/taxallnomy. Instructions to access the data programmatically through API or to produce the Taxallnomy database in a local machine are also available at the Taxallnomy website. Local production is very simple and grants the use of updated information, processed straight from updated NCBI Taxonomy.

## Construction and content

### Data source

Dump files with taxonomic information provided by the NCBI Taxonomy FTP server (ftp.ncbi.nih.gov/pub/taxonomy/) were used for the construction of the Taxallnomy database. Specifically, the dump files containing the parent–child relationship (nodes.dmp) and the taxa names (names.dmp), which are available in both older (taxdump) and newer (new_taxdump) versions of NCBI Taxonomy, were used to generate Taxallnomy tables. The results presented in this work were obtained using dump files downloaded on November 11, 2020, although the Taxallnomy website is kept up to date.

### Concepts

Here we present common terms used when referring to the hierarchical structure of NCBI Taxonomy. The taxonomic tree of NCBI Taxonomy consists of several taxa organized in a hierarchical data structure. All taxa have a name (e.g. *Homo sapiens*, Mammalia, Bacteria) and a numeric identifier (Taxonomy identifier or txid; e.g. 9606 for *Homo sapiens*) associated to, and they correspond to the nodes of the tree. Each taxon is connected to a single node of a level above (parent taxon), except for the root node which is positioned on the top of the tree. Furthermore, a taxon may be connected to one or more nodes of a level below (child taxon); when a taxon is not connected to any child taxon, it is referred to as leaf taxon (or leaf node). Each taxon may or may not (e.g. clade) have one of the 41 taxonomic-ranks assigned to it (Table 1). Taxonomic-ranks also follow a hierarchy such that a taxon of a higher rank cannot be a descendant of a taxon of a lower rank (e.g. a taxon of phylum rank cannot be a descendant of a taxon of class rank). In this work, we also refer to the taxonomic-ranks through numbers which are the taxonomic-rank levels. Therefore, the taxonomic-rank level ranges from 1 to 41, and the highest (Superkingdom) and the lowest (Isolate) taxonomic-ranks have respectively the rank levels 1 and 41. Not all taxa on NCBI Taxonomy have a taxonomic-rank assigned to them and those taxa were referred to as “no rank” (e.g. Tetrapoda, NCBI:txid32523) or more recently some are referenced as “clade”. They are useful because they add phylogenetic separations in the hierarchy. In this work, we will refer to “no rank” plus “clade” as the unranked taxa, since they have useful names and taxonomic IDs attributed but no taxonomic-rank assigned i.e. they are not named after a Class, Order, Family, etc. And we will refer to “missing taxonomic-ranks” to the ones currently lacking in the lineage, which names will therefore be provided by Taxallnomy. A taxonomic lineage, or simply lineage, of a taxon is referred to as the set of nodes in the hierarchical structure which takes the taxon to the top of the hierarchy (in this case, the root node) and it may be composed of both taxonomically ranked and unranked taxa. Along the lineage of a taxon, we obtain the taxonomic-rank classification in each level and might verify that some taxonomic-ranks could be missing (e.g. *Mycrocystis* class). Finally, some taxa in the tree were considered in NCBI Taxonomy as unclassified taxa, which contain the term *unpublished*, *unidentified*, *unassigned*, *environmental samples*, or *incertae sedis* on its name, and therefore their children are not subjects for taxonomic classification, although their parents are.

**Table 1:** Taxonomic-ranks found in NCBI Taxonomy

Level	Name	Abbrev.^a^	Prior.^b^	# of taxa^c^	# of lineages^d^	Level	Name	Abbrev.^a^	Prior.^b^	# of taxa^c^	# of lineages^d^
1	Superkingdom	spKin	1	4	728,071	22	Tribe	Tri	15	2113	158,026
2	Kingdom	Kin	8	11	648,200	23	Subtribe	sbTri	24	497	34,578
3	Subkingdom	sbKin	21	1	46,229	24	Genus	Gen	2	84,642	726,932
4	Superphylum	spPhy	38	1	70	25	Subgenus	sbGen	26	1596	18,815
5	Phylum	Phy	6	149	722,901	26	Section	Sec	30	436	5302
6	Subphylum	sbPhy	9	26	573,559	27	Subsection	sbSec	36	21	314
7	Infraphylum	inPhy	40	0	0	28	Series	Ser	37	9	174
8	Superclass	spCla	20	6	73,676	29	Subseries	sbSer	41	0	0
9	Class	Cla	7	394	715,953	30	Species group	Sgr	27	326	11,709
10	Subclass	sbCla	10	154	306,936	31	Species subgroup	sbSgr	31	124	964
11	Infraclass	inCla	14	18	177,636	32	Species	Spe	3	507,981	726,733
12	Cohort	Coh	16	5	155,939	33	Forma specialis	Fsp	32	714	829
13	Subcohort	sbCoh	29	3	8220	34	Subspecies	sbSpe	25	24,809	29,339
14	Superorder	spOrd	19	55	101,075	35	Varietas	Var	28	8037	8359
15	Order	Ord	5	1518	723,930	36	Subvariety	sbVar	39	5	5
16	Suborder	sbOrd	12	362	220,298	37	Forma	For	34	520	521
17	Infraorder	inOrd	17	130	140,252	38	Serogroup	Srg	35	143	356
18	Parvorder	prOrd	23	25	43,046	39	Serotype	Srt	18	1174	107,446
19	Superfamily	spFam	13	843	191,499	40	Strain	Str	22	42,857	43,064
20	Family	Fam	4	8658	726,481	41	Isolate	Iso	33	641	641
21	Subfamily	sbFam	11	2936	270,677						

^a^Rank name abbreviation

^b^Rank priority order during the rank assignment step

^c^Number of distinct taxa in the rank

^d^Number of leaf lineages with the rank

### Database construction

The algorithmic challenge that we proposed for this work is to fill in the blanks on the taxonomic lineage considering the taxonomic-ranks. To accomplish this, we created an algorithm that performs one of these operations: (1) assigns missing taxonomic-ranks, with the appropriated name and txid to available currently unranked taxa (presently named clades or “no rank” taxa) throughout the taxonomic tree, if possible, or (2) creates nodes to add the missing taxonomic-rank taxa, carefully interpolating them in the hierarchy without affecting it, and naming it accordingly to its child, or even (3) creates children taxa when the lineage does not present them but others do so. In all cases prefixes will distinguish Taxallnomy additions from the original names i.e. no completely original name will be confused with actual taxonomic-rank name, e.g. in the *Homo sapiens* (NCBI:txid9606) lineage, for the sbCla_Theria, the Subclass taxonomic-rank was assigned to the “no rank” Theria (prefix sbCla_); moreover, in Tri_of_*Homo*, the Tribe rank was assigned to an interpolated new node, parent of Genus *Homo* (prefix Tri_of_); furthermore, in sbSpe_in_*Homo sapiens*, the Subspecies rank was assigned to a created node to be a child of species *Homo sapiens* (prefix SbSpe_in_). Therefore, prefixes make reference to the procedure, and thus the created names will differ from NULL in bioinformatics analyses, and will never be mistaken by taxonomy experts, otherwise, they might suggest a position in the tree for putative creation of an actual taxonomic-rank. Moreover, since Taxallnomy has a format of a table, obviously bioinformaticians might make use of only the most commonly used taxonomic-ranks, selecting a few columns to classify the data.

### Procedure for assigning taxonomic-ranks to unranked taxa

**Fig. 1:**
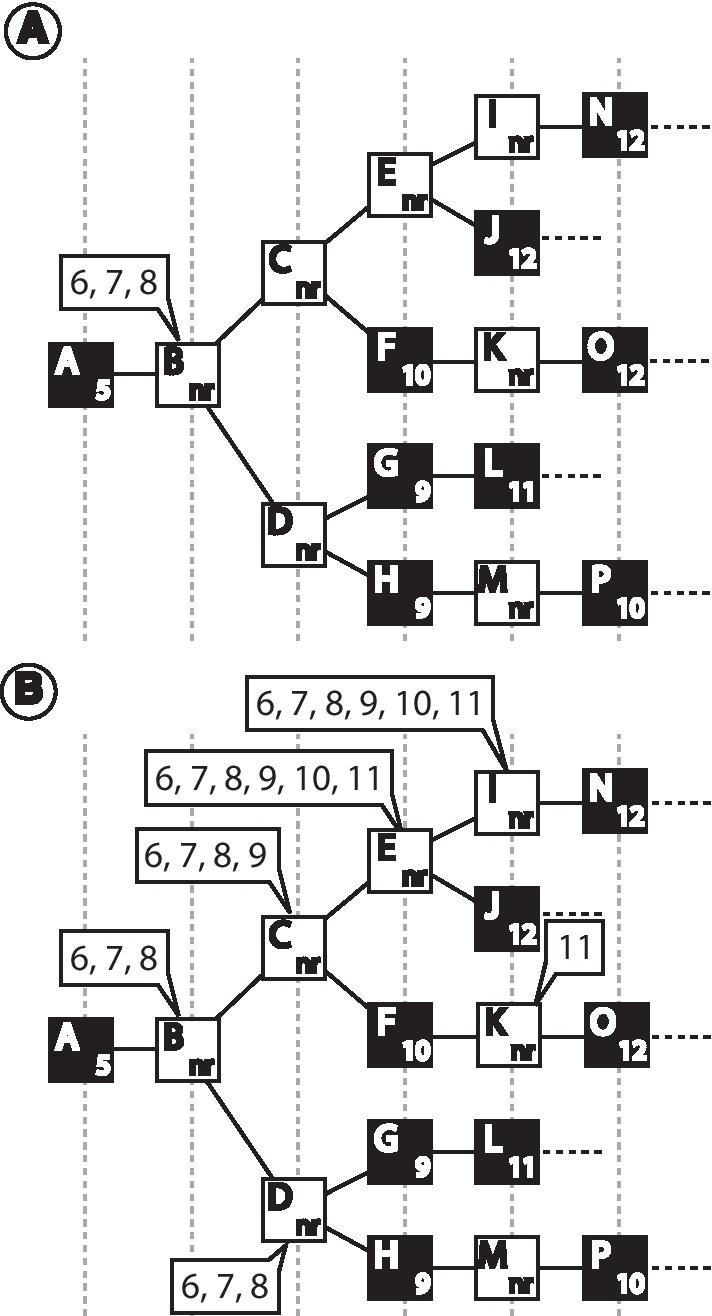
Evaluation of unranked taxa for rank assignment. Ranked nodes are denoted by filled squares. **a** In the hypothetical taxonomic tree, node B is between taxonomic-rank level 5 (phylum) which is the lowest rank among its ascendant nodes; and taxonomic-rank level 9 (class) which is the highest taxonomic-rank among all its descendant nodes. Thus, the taxonomic-ranks that node B could assume without affecting the rank hierarchy are subphylum (6), infraphylum (7), or superclass (8), in the balloon. **b** After evaluating all unranked taxa, we can find (i) those unranked taxa that cannot assume any ranks (node M, since it is between ranked nodes 9, class, and 10, subclass) and (ii) those that could assume one or more ranks (nodes with a balloon)

The first approach is to map existing nodes that are unranked taxonomically to assign taxonomic-ranks to them, which will append the prefix Cla_, Ord_, Fam_, etc. The algorithm begins evaluating all unranked taxa by moving through the hierarchical levels of the taxonomic tree. For each unranked taxon found, the algorithm evaluates if some of the 41 taxonomic-ranks occurring in NCBI taxonomy (Table 1) can be assigned to it. Since the taxonomic-ranks follow a hierarchy, an unranked taxon can assume neither a rank with a level that is lower or equal than those found among its ascendant nodes nor a rank with a level that is higher or equal than those found among its descendant nodes. So, to determine the ranks that an unranked taxon could be assigned with, the algorithm firstly verifies the highest and the lowest taxonomic-rank levels found among its ascendant and descendant nodes, respectively. The rank levels that are between them are those that can be assigned to the unranked taxon in conformation with the rank hierarchy, thus considered as candidate ranks (numbers in balloons in Fig. 1).

**Fig. 2:**
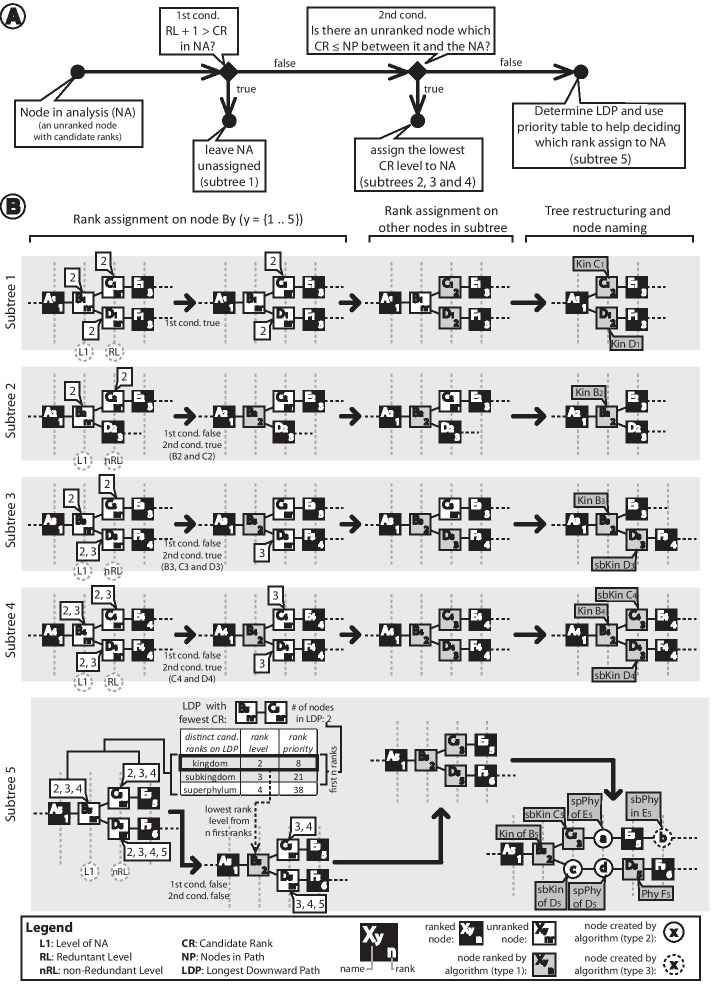
Rank assignment step. See text for a detailed explanation. **a** Set of rules followed by the algorithm to assign a single rank to an unranked taxon with candidate ranks. **b** Some examples of complex situations faced by the algorithm and the way it solves the rank assignment, the tree restructuring, and the node naming. Candidate ranks of an unranked taxon are in balloons. Square nodes represent taxa originally found in NCBI Taxonomy. Names assigned to nodes ranked (type 1) or created (types 2 and 3) by the algorithm are shown in gray balloons

After evaluating all unranked taxa, the algorithm proceeds to the rank assignment procedure. In this step, the algorithm goes through the taxonomic tree, starting from the root, looking for unranked taxa with candidate ranks to assign an appropriate rank to it. A simple case of this assignment occurs in unranked taxa that have a single candidate rank without any unranked taxon as its parent or child (e.g. node K in Fig. 1b). In this case, the algorithm simply assigns the candidate rank to the taxon. The assignment process becomes more complex when the unranked taxon has two or more candidate ranks and/or has additional unranked taxon among its child nodes since it enables more than a single valid way to perform the rank assignment. To deal with those situations, we created a set of algorithmic rules to decide the nodes and the taxonomic-ranks to be used for the assignment (Fig. 2a). The rules were designed aiming to assign ranks to as many unranked taxa as possible while prioritizing the assignment of those ranks most frequently found in the lineages of the taxonomic tree.

For a better understanding of the assignment rules, consider the subtrees in Fig. 2b which illustrates different situations found by the algorithm for the rank assignment problem. In all subtrees, the node in analysis (NA) is the unranked taxon B_n_ (n = {1, 2,…,5}). Also, the hierarchical level of the NA is referred to as the first level (L1). The first condition evaluated by the algorithm is the existence and the number of levels that are redundant with the L1 (RL). We consider that levels following the L1 are redundant if all nodes on it are (1) unranked and (2) have the same candidate ranks as the NA, and if (3) the level above it is the L1 or a redundant level without leaf nodes. If the number of candidate ranks (CR) in the NA is less than the number of levels that need a rank (L1 plus consecutive redundant levels), there are not enough ranks to assign to the nodes on those levels. In this case, one option is to assign the lowest rank level among the candidate ranks to the NA and leave some of its descendant nodes unranked. However, we opted to leave the NA without a rank so that the unranked taxa of further levels could have a rank assigned. This procedure was chosen as this could result in a more unranked taxa with a rank assigned, favoring the dichotomization of the final tree. In subtree 1 (Fig. 2b), the NA (B_1_) has one candidate rank (taxonomic level 2), and the level following the L1, which is composed of nodes C_1_ and D_1_, meets all conditions established to be a redundant level (RL). Since the number of candidate ranks in the NA (CR = 1) is not sufficient to rank the nodes on L1 and further redundant levels (L1 + RL = 2), the algorithm leaves the node B_1_ without a rank, allowing the nodes of further level (C_1_ and D_1_) to have a taxonomic-rank assigned.

If the previous condition is not true then the next condition evaluated by the algorithm is the existence of unranked taxa in the subtree in which the number of candidate ranks on it is equal to or less than the number of consecutive unranked taxa in the path between it and the NA. If a node in this condition is found (Fig. 2b, subtrees 2, 3, and 4), all candidate ranks on it could likely be distributed along with the unranked taxa in the path linking it to the NA. So, in this condition, the algorithm assigns the lowest rank level among the candidate ranks to the NA.

If none of those conditions apply, it is an indicator that the subtree cannot bear to assign all candidate ranks to its unranked taxa (Fig. 2b, subtree 5). In this case, the algorithm has to decide the taxonomic-ranks to be used for the assignment process. To help with this, we determined an order of priority of which taxonomic-ranks should be firstly assigned based on the frequency that they appear in the leaf lineages (Table 1). The more frequent the rank, the higher is its priority. To make use of this order of priority, the algorithm searches for the longest downward path (LDP) of consecutive unranked taxa, starting from the NA. Once the LDP is found, the algorithm stores the number of nodes on this path and the distinct candidate ranks found among the nodes comprising the path. If there is more than one LDP in the subtree, the algorithm considers the one with less distinct candidate ranks along the path. Then, the candidate ranks are sorted according to their order of priority and the first *n* ranks, in which *n* is the number of nodes in the LDP, are extracted. The extracted ranks will be those to be assigned to the nodes in the LDP. Since the NA is the first node in the LDP, the algorithm picks the rank of the lowest level among the extracted ranks and assigns it to the NA.

After an unranked taxon has a rank assigned, the further unranked taxa have their list of candidate ranks updated and visited by the algorithm to perform the same analysis. After performing this procedure on all unranked taxa, all of them will have a single rank or no rank assigned (Fig. 2b).

### Making the tree complete hierarchically

The final step of the algorithm consists of creating and deleting nodes to make the taxonomic tree complete, hierarchically, since some taxonomic-ranks were not yet made present by the procedure of assigning taxonomic-ranks to unranked taxa described above; and on defining a name for the unranked taxa and its corresponding created nodes (Fig. 2b, tree restructuring and node naming step). For this, the algorithm will delete all unranked taxa that did not have a taxonomic-rank assigned in the previous procedures; as occurred with the nodes “B_1_”, “C_2_”, and “C_3_” (Fig. 2b, subtrees 1, 2, and 3). On the other hand, unranked taxa with a rank assigned are maintained and new names are assigned to them to indicate that they were originally unranked. The new names of these nodes consist of the abbreviation of the rank assigned (Table 1) followed by the original name of the node; i.e. in the node “C_1_” (Fig. 2b, subtree 1), since it has the Kingdom rank assigned, its new name will be “Kin C_1_”. The nodes that meet this condition are referred to as taxa of type 1. An example of a node of this type in the Taxallnomy tree is sbCla_Theria, which is the proposal for the human subclass.

The taxonomic tree has portions where two consecutive taxa do not have consecutive ranks. In this case, the algorithm creates nodes between them and assigns the created nodes with ranks that are missing. For instance, we could observe in subtree 5 of Fig. 2b that there should be nodes with ranks of Superphylum (level 4) between the nodes “C_5_” (Subkingdom, level 3) and “E_5_” (Phylum, level 5). To fulfill this gap, the algorithm creates between them a node (node “a”) with Subphylum rank assigned to it. This type of node is referred to as type 2 and is named using the abbreviation of the assigned rank followed by the preposition “of” and the original name of its first ranked descendant node. For the node “a”, since it has the node “E_5_” as the first ranked descendant node, it is named as “spPhy of E_5_”. Human’s tribe, for example, is proposed to be Tri_of_Homo, which Homo is a Genus stated in the original database.

Finally, if there are some lineages with missing ranks because there is no node of a higher level, the algorithm will also visit these lineages and create a node for each missing rank. In subtree 5 of Fig. 2b, the node “E_5_” is a leaf node of Phylum rank (level 5). Since “E_5_” is a leaf node, all ranks after Phylum are missing in this lineage. In this case, Taxallnomy will visit these nodes and create nodes to fulfill those missing ranks. The node “b” in subtree 5 (Fig. 2b) is a node created for this purpose. To name this node, the algorithm takes the abbreviation of the missing rank followed by the preposition “in” and by the original name of the last taxon of the lineage (“sbPhy in E_5_”). These nodes are referred to as taxa of type 3. They are useful in cases when there are, for example, subspecies declared in the database. For instance, *Sus scrofa* (NCBI:txid9823) has over 60 thousand proteins deposited, but only around 1.5 thousand are assigned to one of its 11 subspecies. Therefore, most of those entries are “NULL” for the Subspecies rank in the original database; but, by creating the node of type 3, all of them are treated to have a node of Subspecies rank named “sbSpe in Sus scrofa”. Another usage of nodes of type 3 is on metagenomics analysis (Fig. 7), when there are entries annotated with taxa of lower rank levels and one wants to count the number of distinct taxa of higher rank levels.

### Rules for assigning species or genus ranks

Species and Genus are ranks with high frequency (Table 1), thus both have high priority during the rank assignment procedure. Therefore, an unranked taxon that has one of those ranks as candidates are more likely to have one of them assigned. We evaluated some lineages of leaf taxa lacking for Species or Genus ranks and verified that some unranked taxa are appropriate to have one of those ranks. For instance, in an older version of NCBI Taxonomy (September 19, 2016), *Beringia wynnei* (NCBI:txid1037071) was a leaf taxon of Species rank that did not have a Genus rank in its lineage. However, its lineage contained an unranked taxon named *Beringia* (NCBI:txid1037069), which had the Genus rank appropriately assigned by the algorithm. Similarly, *Nocardia argentinensis* ATCC 31,306 (NCBI:txid1311813), in the same version of NCBI Taxonomy, was a “no rank” leaf taxon, which did not have a node with Species rank in its lineage, but it contained an unranked taxon named *Nocardia argentinensis* (NCBI:txid1311812). The current algorithm also appropriately assigned the Species and Subspecies ranks to the nodes *N. argentinensis* and *N. argentinensis* ATCC 31306, respectively. However, depending solely on these rules incurs some obvious errors, like in those unranked leaf taxa which do not have nodes with Species and Genus rank in its lineage. For instance, Rosodae (NCBI:txid721787) is a “no rank” leaf taxon that has a parent node with Subfamily rank (level 21). According to the algorithm, Rosodae could have ranks ranging from Tribe (level 22) to Isolate (level 41), and, based on the rank priority, it would be assigned to Genus rank, which is not a proper rank for it. To correct this situation, special rules were added to the algorithm to have the Species and Genus ranks assigned to an unranked taxon. We established that the Species rank assignment to an unranked taxon should occur only if, among its ascendant nodes, there is a node of Genus rank in the original database. On the other hand, an unranked taxon should have the Genus rank assigned if there are nodes of Species rank among its descendants in the original database. Moreover, the assignment of both ranks to an unranked taxon should not occur if a node has terms in its name that identify it as an unclassified entry. With these rules, the unranked taxon Rosodae mentioned before has the Tribe rank assigned instead of Genus rank.

### The identifiers for taxa created/modified

The primary identifier of each node comprising the Taxallnomy tree is the Taxonomy ID provided by the NCBI Taxonomy database. However, since the Taxallnomy algorithm assigns ranks to nodes and creates new nodes, we formulated a code that properly identifies them. The Taxallnomy code consists of three digits added as a decimal number in the Taxonomy ID of each node. The first two digits indicate the taxonomic-rank in to which it was assigned. It goes through the code "01" to "41", in which the first code ("01") refers to the Superkingdom rank and the last one ("41") refers to the Isolate rank. The third digit ranges from 1 to 3 and indicates the approach used by the algorithm to create/modify a node. The codes 1, 2, and 3 refer respectively to taxa of type 1, type 2, and type 3. For instance, in the taxon code 6072.031, 6072 corresponds to the NCBI Taxonomy ID (Eumetazoa) and 031 is the code added by the Taxallnomy algorithm, indicating that it is a node of type 1 created on Subkingdom rank. Using the Taxallnomy name convention, the name of this node will be “sbKin Eumetazoa”. Furthermore, taxa originally ranked in the NCBI Taxonomy database has the code 000 included (e.g. 9606.000, which stands for the species *Homo sapiens*).

### Usability and availability

Users can query the Taxallnomy database and download the results using its web interface at http://bioinfo.icb.ufmg.br/taxallnomy. In the web interface, users can also find an interactive Taxallnomy tree, which allows easy exploration of its hierarchical structure. Advanced users can also programmatically query the Taxallnomy database using our REST service for this database (see the Taxallnomy web page for more instructions). For experiencing Taxallnomy, one can access the website and add, for example, this list of seven Genus-TxIDs: 9030, 8500, 8507, 28376, 8468, 643744, 436494; or type and add, one by one, their taxon names: *Gallus*, *Crocodylus*, *Sphenodon*, *Anolis*, *Chelonia*, *Brachylophosaurus*, and *Tyrannosaurus*. This will generate the complete hierarchical subtree comprising those taxa in which, as one reads this, some order and class ranks might be missing yet. It is worth mentioning that those unranked taxa which had no rank assigned by the algorithm could also be displayed in the tree, demonstrating that Taxallnomy does not harm the NCBI Taxonomy hierarchy.

Users with high demand can also find all necessary files to have a copy of the Taxallnomy database in a local MySQL database at the Taxallnomy SourceForge page (https://sourceforge.net/projects/taxallnomy). The Taxallnomy database comprises five main tables named “lin”, “lin_name”, “tree_complete”, “tax_data”, and “rank”. The first two tables have the taxonomic lineages that comprise the Taxallnomy tree. The tables have a column containing the NCBI Taxonomy ID (txid), which is the primary key column of the tables; and 41 columns representing the 41 taxonomic-ranks found in the NCBI Taxonomy database. In the “lin” table, the taxonomic-rank columns are filled with taxonomic codes, whereas, in “lin_name, those columns are filled with taxonomic names. The table “tree_complete” contains all parent–child relationships in the Taxallnomy database that make the hierarchical structure complete. Two other hierarchically incomplete versions of the tree table are also available in the Taxallnomy data source; one is the table “tree_all”, which includes the unranked taxa that did not have a rank assigned, and the other is the table “tree_original”, which has the same hierarchical structure as the one provided by the NCBI Taxonomy database. In the “tax_data” table, users can find information about each taxon comprising the tree, such as its scientific name, common name, and rank level. Finally, the “rank” table contains information about the ranks comprising the taxonomic tree, such as name, level, priority order, and abbreviation.

Since the NCBI Taxonomy database is frequently updated, the database used in the Taxallnomy web page and provided in its SourceForge page is updated weekly. Users with a local copy of the Taxallnomy database can acquire the updated database from its SourceForge page. Alternatively, we also provide a Perl script with the Taxallnomy algorithm implemented at https://github.com/tetsufmbio/taxallnomy. The script can be executed in a UNIX system with an internet connection, which is required for downloading the latest version of the NCBI Taxonomy database. Users can also execute the script by providing a local copy of the compressed dump files provided on the NCBI Taxonomy FTP server.

## Utility and discussion

### Taxallnomy overview

**Fig. 3:**
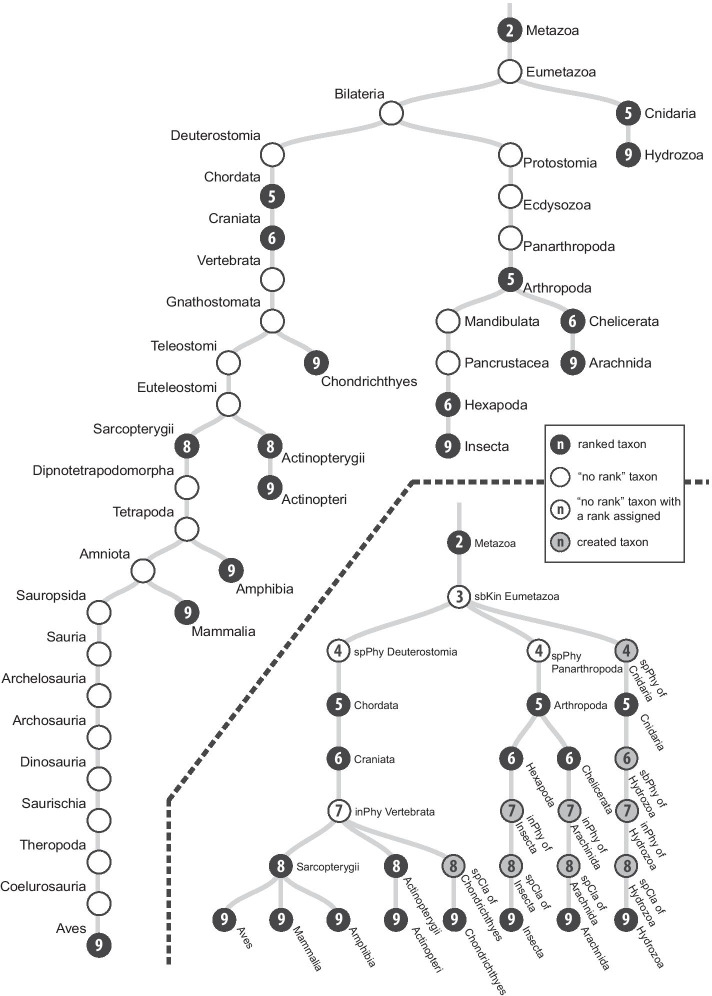
Subtree of the taxonomic trees comprising some taxa of the Class rank of Kingdom Metazoa. The upper tree is from NCBI Taxonomy and the lower tree is from Taxallnomy databases. The number in the node (n) indicates the taxonomic-rank level (see Table 1)

The new taxonomic database was named Taxallnomy since it provides names for all ranks that are missing in a taxonomic lineage. To exemplify this, we took a portion of the taxonomic tree comprising some Classes of Kingdom Metazoa (Fig. 3). Note that in the tree currently provided by NCBI Taxonomy (Fig. 3, upper tree) some ranks are absent (e.g. Superclass for Insecta) and some taxa do not have a rank in a taxonomic lineage (e.g. Eumetazoa, Bilateria). By taking the equivalent portion of the tree from the Taxallnomy database (Fig. 3, lower tree) we could observe that all taxa with the same rank are positioned in the same hierarchical level. To achieve this, the Taxallnomy algorithm assigned ranks to some unranked taxa, such as Eumetazoa (level 3), Deuterostomia (level 4), and Panarthropoda (level 4); deleted others, such as Bilateria, Vertebrata, and Gnathostomata; and created nodes to fill the missing ranks in a lineage, such as “spPhy (Superphylum) of Cnidaria”, “sbPhy (Subphylum) of Hexapoda”, “spCla (Superclass) of Chondrichthyes” and others. Observing the exemplified portion of the Taxallnomy tree in more detail, one could question why the algorithm ranked the taxa Deuterostomia and Panarthropoda to Superphylum (level 4) instead of ranking the taxon Bilateria. Another questionable point can be found in the lineage of Insecta in which the algorithm did not rank the taxa Mandibulata or Pancrustacea to Subphylum (level 6), but created a new node (sbPhy of Hexapoda) instead. All those ranking patterns executed by the algorithm were established to agree with the rank hierarchy. The taxon Bilateria could not have the rank Superphylum assigned because one of its descendant taxa (Scalidophora—not shown) has this rank. Similarly, Mandibulata and Pancrustacea could not have the rank Subphylum assigned because both taxa have descendant taxa (e.g. Crustacea—not shown) with this rank.

Taxallnomy database consists of a total of 9,875,550 nodes, in which 9,183,606 (92.99%) of them are nodes created by the Taxallnomy algorithm or unranked taxa that had a rank assigned. Among them, 170,742 (1.86%) are of type 1, 4,225,358 (46.01%) of type 2 and 4,787,506 (52.13%) of type 3. Moreover, the number of unranked taxa used to create the nodes of type 1 corresponds to 99.85% of all unranked taxa found in the original tree (170,991 nodes). The number of leaf taxa totalized 728,071, of which 68.18% are from Eukaryota, 8.51% from Bacteria, 0.13% from Archaea, and 23.38% from Virus or Viroids Superkingdoms. In these counting, unclassified taxa (including unpublished, unidentified, unassigned, environmental, or *incertae sedis* taxa) were not included.

**Fig. 4:**
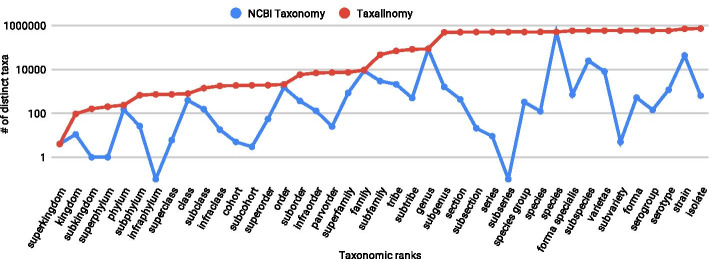
Number of distinct taxa in NCBI Taxonomy and Taxallnomy databases along the taxonomic-ranks

Since the Taxallnomy tree is hierarchically complete and consequently all taxonomic lineages have all nodes of each rank level, the number of distinct taxa found in each rank expectedly increases as we go through the ranks (Fig. 4). This contrasts with the original tree from the NCBI Taxonomy database, which shows a wide fluctuation in the number of distinct taxa along with the ranks. The contribution of the Taxallnomy database in creating nodes and names can be noticed by measuring the differences in the number of distinct taxa on each taxonomic level on both trees.

**Fig. 5:**
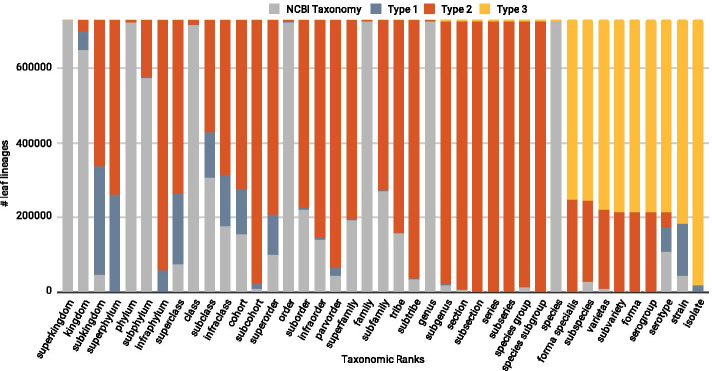
Frequency of taxa originally ranked or modified/created along the taxonomic lineages. A total of 728,071 taxonomic lineages of leaf taxa were evaluated

We could also observe the contribution of Taxallnomy in completing the hierarchical structure by accounting for the created/modified nodes comprising the lineages of leaf taxa (Fig. 5). Disregarding the main ranks (Superkingdom, Phylum, Class, Order, Family, Genus, and Species), which are found in almost all lineages, most of the ranks are found originally in few lineages and had a node included by the Taxallnomy algorithm. Nodes of type 1 are found mainly in the first ranks (from Kingdom to Superorder ranks) and lower ranks (Serotype to Isolate), indicating the existence of unranked taxa in those ranges on the original tree worthy to be ranked. Taxonomic lineages exhibit a great amount of type 2 nodes on ranks higher than Species rank level and that are not part of main ranks. This occurs because there are no or few unranked taxa to assign a rank in those ranges, which forces the algorithm to create new nodes in the original tree. Finally, the nodes of type 3 are concentrated in the lowest ranks (from Forma specialis to Isolate ranks). This indicates that many leaf taxa analyzed are from Species rank, causing the algorithm to create taxa of type 3 for the further ranks.

### Application cases

The lack of a taxon on a specified rank in a lineage could be inconvenient for any analysis in which we ask something about the taxonomic-ranks on our data. One could take a simple BLAST result and ask which taxa from a specified rank are found among the subjects retrieved. If one tries to answer this using the original data from the NCBI Taxonomy database, he could come across subjects belonging to species that do not have a taxon with the queried rank. In this situation, we could take advantage of the Taxallnomy database, which has the gaps of all taxonomic lineage fulfilled. For instance, taking a BLAST [20] result that used the human P53 protein as a query against the UniProt database [10] (Table 2), we could observe that most of the subjects retrieved in this analysis belong to organisms that have a taxon with Class rank (Mammalia, Coelacanthimorpha, Aves, Amphibia, and Actinopteri) in the original database, but some of them have a Class rank created by Taxallnomy (“Cla_of_Crocodylia” and “Cla_of_Testudines”). Without this information, we could not have an idea if those subjects are from organisms of the same Class or not. If we consider now the Superorder rank, we could observe that eight subjects belong to organisms that lack this rank in their lineage. By fulfilling those spots with information from Taxallnomy, we have the eight organisms classified in four distinct Superorders (“spOrd_of_Passeriformes”, “spOrd_of_Psittaciformes”, “spOrd_of_Crocodylia”, and “spOrd_of_Testudines”).

**Table 2:** BLAST result with taxonomic data from Taxallnomy

Entry	E-value	Ident (%)	txid	Class^a^	Superorder^b^	Species
P10360	0	100	9031	Aves	Galloanserae	*Gallus gallus*
A0A674GK28	1E−161	73.3	59729	Aves	spOrd_of_Passeriformes	*Taeniopygia guttata*
A0A672TYH0	1E−152	82.9	2489341	Aves	spOrd_of_Psittaciformes	*Strigops habroptila*
A0A1U7SJ11	1E−135	59.2	38654	Cla_of_Crocodylia	spOrd_of_Crocodylia	*Alligator sinensis*
A0A2U4C2U9	3E−133	55.4	9739	Mammalia	Laurasiatheria	*Tursiops truncatus*
A0A151MW63	4E−133	58.6	8496	Cla_of_Crocodylia	spOrd_of_Crocodylia	*Alligator mississippiensis*
A0A6J3QPS7	5E−133	55.4	9739	Mammalia	Laurasiatheria	*Tursiops truncatus*
A0A3Q0FUE5	1E−132	66.4	38654	Cla_of_Crocodylia	spOrd_of_Crocodylia	*Alligator sinensis*
A0A2F0B4V5	3E−132	55.1	9764	Mammalia	Laurasiatheria	*Eschrichtius robustus*
A0A341BQX3	1E−131	54.9	1706337	Mammalia	Laurasiatheria	*Neophocaena asiaeorientalis*
A0A340XCN5	1E−131	54.9	118797	Mammalia	Laurasiatheria	*Lipotes vexillifer*
A0A455C1G1	1E−131	54.9	9755	Mammalia	Laurasiatheria	*Physeter macrocephalus*
A0A6A1Q3Q7	1E−131	54.9	9770	Mammalia	Laurasiatheria	*Balaenoptera physalus*
Q8SPZ3	8E−131	54.6	9749	Mammalia	Laurasiatheria	*Delphinapterus leucas*
A0A2Y9Q793	8E−131	54.6	9749	Mammalia	Laurasiatheria	*Delphinapterus leucas*
A0A4V5P9N3	8E−131	54.6	40151	Mammalia	Laurasiatheria	*Monodon monoceros*
A0A383YUA7	1E−130	55.4	310752	Mammalia	Laurasiatheria	*Balaenoptera acutorostrata*
A0A484GHZ0	2E−130	54.1	103600	Mammalia	Laurasiatheria	*Sousa chinensis*
K7G3P4	2E−130	53.4	13735	Cla_of_Testudines	spOrd_of_Testudines	*Pelodiscus sinensis*
P41685	3E−130	55.6	9685	Mammalia	Laurasiatheria	*Felis catus*
A0A218U9L5	1E−129	86.2	299123	Aves	spOrd_of_Passeriformes	*Lonchura striata*
A0A452GW06	2E−129	55.8	38772	Cla_of_Testudines	spOrd_of_Testudines	*Gopherus agassizii*
A0A6G1A9W5	4E−129	55.5	9678	Mammalia	Laurasiatheria	*Crocuta crocuta*
A0A671EZ36	6E−129	56.6	59479	Mammalia	Laurasiatheria	*Rhinolophus ferrumequinum*
A0A2K5TIV2	6E−129	55.3	9685	Mammalia	Laurasiatheria	*Felis catus*

^a^Taxa of Class rank created by Taxallnomy begin with “Cla_”

^b^Taxa of Order rank created by Taxallnomy begin with “spOrd_”

**Fig. 6:**
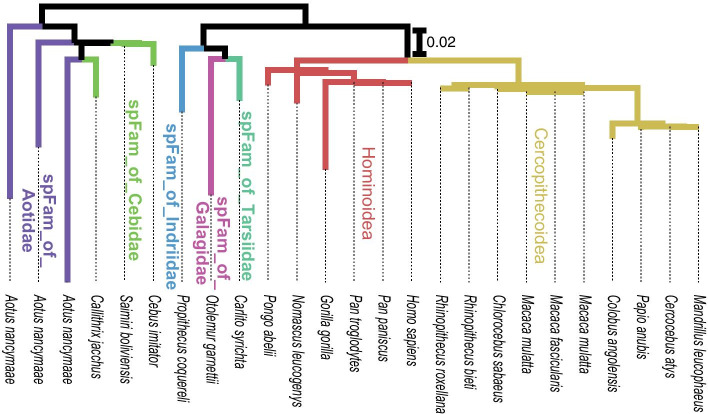
Phylogenetic tree of tumor protein P53 of species from Order Primates. Branch color evidences the distinct taxa of Superfamily rank comprising the tree. Superfamilies beginning with “spFam_of_” (in bold) are taxa created by the Taxallnomy algorithm

Similarly, taxonomic data are frequently incorporated into a phylogenetic tree to evidence some taxonomic groups. By embedding taxonomic data from Taxallnomy to a phylogenetic tree, a user can select a rank and evidence taxa comprising the selected rank without worrying about the missing ranks. We exemplify this by evidencing the distinct Superfamilies comprising a phylogenetic tree generated using the “tumor protein 53” sequences of species from Order Primates (Fig. 6). In this tree, we could evidence five taxa that were created by Taxallnomy: spFam_of_Tarsiidae, spFam_of_Galagidae, spFam_of_Indriidae, spFam_of_Cebidae, and spFam_of_Aotidae.

Metagenomics analyses heavily rely on taxonomic data and information about taxonomic-ranks. After the taxonomic annotation performed by software like MEGAN [21], MG-RAST [22], or the pipeline from EBI Metagenomics [23], researchers in this field seek a metagenomics profile to verify which taxa are predominant in an environmental sample. Since taxonomic annotation performed by those programs is based on the NCBI Taxonomy database, the taxonomic profile is usually performed by firstly extracting the taxonomic lineages of those taxa that were assigned to a read and then plotting the abundance of taxa in each taxonomic-rank separately. However, as stated initially, some ranks are missing in some taxa, which obliges us, in the end, to include all those taxa without rank in a separate group (e.g. unclassified) or to omit them in the graphic representation. The same procedures are taken in the case in which there is a read annotated to a taxon of lower rank level, and we want to have a taxonomic profile of a higher rank level, e.g. reads that could be annotated only as Proteobacteria, a taxon of rank Phylum, would not be counted in the following ranks (class, order, and so on). An alternative representation of the taxonomic profile is to show the taxa abundance along the taxonomic tree without accounting for the taxonomic-rank. The advantage of this approach is that abundance analysis is performed in all available nodes (ranked or unranked) of the taxonomic tree. However, since the depth of taxonomic lineages could vary between taxa, e.g. bacterial species *Escherichia coli* (NCBI:txid562) and *Microcystis aeruginosa* (NCBI:txid1126) have eight and 10 taxa on their lineages, respectively, same taxonomic-ranks, even the species rank (the only natural rank), might be displayed in different level of the profile.

**Fig. 7:**
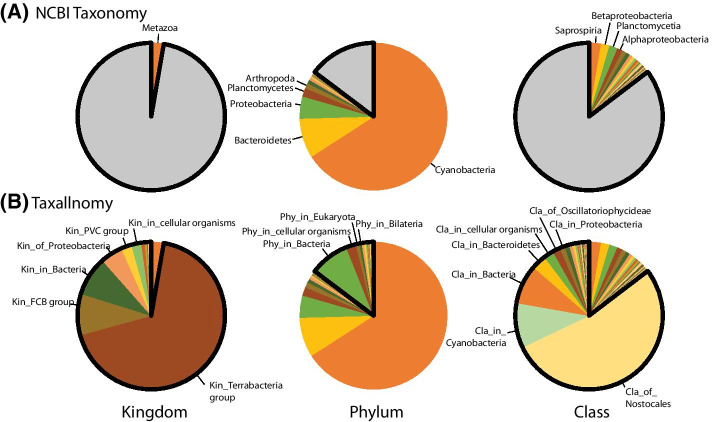
Taxonomic profiles of the metagenomic sample from a tropical freshwater reservoir in Brazil. Taxonomic profiles were generated at Kingdom, Phylum, and Class levels by using taxonomic sources from **a** NCBI Taxonomy and **b** Taxallnomy databases. For the taxonomic profiles obtained using NCBI Taxonomy, the gray portion of the chart comprises all sequences annotated to taxa that are “NULL” for those ranks in their lineages. These sequences can be further annotated using the lineages retrieved from the Taxallnomy database (in bold). ProjectID of the metagenomic sample on MG-RAST:mgp13799

All these issues can be resolved by using a hierarchically complete taxonomic tree provided by the Taxallnomy database. To exemplify this, we took a metagenomics sample collected from a tropical freshwater reservoir in Brazil (projectID on MG-RAST:mgp13799) and generated its taxonomic profiles using taxonomic sources from NCBI Taxonomy and Taxallnomy. For this, we submitted the reads to MEGAN for taxonomic annotation and retrieved the taxonomic lineage from both databases for each taxon that appeared in the annotation process. Then, we assembled all taxonomic lineages retrieved in a spreadsheet and generated, for instance, pie charts for the ranks Kingdom, Phylum, and Class (Fig. 7). In the profile obtained using NCBI Taxonomy (Fig. 7a), depending on the metagenomic sample and taxonomic-rank in analysis, several reads would be omitted or grouped in the unclassified group since the lineage of the taxa assigned to them miss for those ranks. Since lineages retrieved from the Taxallnomy database have those missing ranks fulfilled, all reads will be considered in the resultant taxonomic profiles (Fig. 7b). Even those reads that had taxa of lower rank levels assigned (e.g. Cellular organisms, Bacteria) can be considered in profiles of higher rank levels through nodes of type 3 created by the Taxallnomy algorithm (e.g. “Phy_in_Cellular organisms”, “Phy_in_Bacteria”). It is worth mentioning that the Kingdom rank is not typically applied in the metagenomic profile since there are no bacterial species cataloged in the NCBI Taxonomy with a taxon of this rank in their lineage. However, since some of them have no rank taxa which had the Kingdom rank assigned to by the Taxallnomy (e.g. PVC group, FCB group, Terrabacteria group), displaying this unusual rank in the profile ends up adding grouping information provided by those no rank taxa. In a typical metagenomic profile, this information would have been lost since no rank taxa, in practice, are discarded from the analysis.

## Discussion

Taxonomy has an extensive history that begins from Aristotle (for review, see [24–27]) and, since then, several approaches have been proposed to classify and name biodiversity. In general, taxonomic databases have two fundamental functions: (1) provide an efficient system of storage and retrieval of taxonomic data; and (2) provide the evolutionary and diversity scenario of the organisms [28, 29]. To meet one or both functions, two approaches prevail in the current taxonomic databases: (1) the rank-based classification, which groups organisms in categories of Linnaean system (Kingdom, Phylum, Class, etc.); and (2) clade-based classification, which names monophyletic clades of a phylogenetic tree. Since both classification systems meet very well one of the functions of taxonomy described above (rank-based approach is more practical and clade-based approach is more explanatory) [29], the use of either methodology is a theme under great debate among taxonomists [30–32].

The main criticism faced by the rank-based classification is the lack of an absolute definition of each rank since there are no well-established criteria for the rank assignment process [33]. For this reason, taxa of the same rank are not necessarily comparable and do not make assumptions about equal age [34, 35], although there are some attempts to make them comparable, by using the temporal banding approach [36–38] or time clips [39]. Despite the inconsistency, taxonomic-ranks still have important roles in facilitating communication [40]. Many regionals, national, or global taxonomic databases follow the taxonomic backbone provided by the Catalogue of Life (CoL), a rank-based global standard taxonomic database built from the consensus classification of more than 3,000 taxonomist expert opinions [41]. Even taxonomic databases that adopt the clade-based approach still maintain the taxonomic-ranks to serve as references [42, 43]. Taxonomic-ranks also provide meaningful information for evolutionary comparison [40, 44]. For example, once we know that *Homo sapiens* is placed in the family Hominidae, we could assert that *Homo sapiens* is more closely related to any species within this family than to any other species which is not Hominidae.

Taxonomic information provided by NCBI Taxonomy [8] is a valuable resource in several bioinformatics fields. Its classification system is a conciliation of both rank- and clade-based approaches. Several tools and software, which have this database as the main subject, have been developed so far either to assist its data retrieval [45–47] and visualization [48] or to improve the hierarchical structure by correcting misclassified organism [49, 50] or by disambiguating taxonomic names for text mining [51–54]. Although the NCBI Taxonomy database has a long life span (since 1991), several reports document challenges presented by the lack of a complete hierarchical rank classification [49, 51, 55–58]. This motivated us to develop Taxallnomy, a database that provides a completely hierarchical NCBI Taxonomy rank classification. By adding new ranked taxa or assigning a rank to a clade, Taxallnomy aggregates the benefits of the taxonomic-ranks present, but not completely, in the taxonomic tree of NCBI Taxonomy. It is important to emphasize that this work is not meant to propose a new systematic approach for taxonomic classification, but an extension of the broadly used NCBI Taxonomy to facilitate the computational use of the rank-based classification on some bioinformatics approaches.

Since the Taxallnomy algorithm could create new nodes (nodes of type 2 and 3) or assign a rank to a preexisting one (nodes of type 1) along with the hierarchical structure, another task performed by the algorithm is to create adequate names for those nodes. Besides the existence of a nomenclature rule to name taxa of a given rank, it would be a complex task to adopt them, since different taxonomic groups have different nomenclature rules [59–61]. So, we established generic rules which take advantage of preexisting names and allow easy identification of the rank and the modifications performed by the algorithm in the hierarchical structure.

There is no comprehensive method to address the problem of the lack of a complete hierarchical rank classification, but some solutions have been practiced. The most common and simplest one is the elimination of the “no rank” taxa throughout the lineage [49, 51, 56]. More sophisticated solutions fill the missing ranks by taking the taxon name of the first taxon of a lower [58] or a higher rank level [57]. These solutions are similar to the procedures used by the Taxallnomy algorithm to create the nodes of types 2 and 3. Type 2 nodes are created whenever there is no node between two taxa of non-consecutive ranks and take the name of the first ranked taxon of the higher rank level as in [57]. The preference to take the name of a higher rank level taxon instead of the lower one is conceptual. For instance, if we have a node “X” of a Phylum rank that has two child nodes “Y” and “Z”, both of the Superclass rank, the Subphylum rank is missing on both Y and Z lineages. By taking the name of the node of the lower rank level (node X) to name the missing rank, both Y and Z nodes would have the same Subphylum (“sbPhy of X”). On the other hand, by taking the node of higher rank level (nodes “Y” and “Z”), both nodes will be in distinct Subphylums (“sbPhy of Y” and “sbPhy of Z”). In theory, we do not know if those lineages are actually of the same Subphylum, so, it would be preferable to separate them into different Subphylums instead of putting them in the same group. The node of type 3, on the other hand, is created whenever a lineage lacks higher rank levels. The algorithm takes the name of the last node of the lineage similarly to [58] since there is no other reasonable taxon in which we could take advantage to name the new node.

Besides the creation and nomination of new nodes based on ranked taxa, a remarkable feature performed by the Taxallnomy algorithm is the rank assignment of a “no rank” taxon. Taxa with “no rank” status are spread throughout the tree and usually are discarded by users or software that require a hierarchically complete tree, which results in the loss of information. In this work, we show that we could take advantage of the “no rank” taxa to fulfill lineages with missing ranks and assist in generating a completely hierarchical taxonomic tree. It is worth mentioning that keeping the “no rank” taxa in the final tree as many as possible is important to preserve the groups already structured by the taxonomic tree. Thus, the current algorithm performs the rank assignment procedure to assign ranks to as many “no rank” taxa as possible. By this, the algorithm has assigned a rank to more than 99% of all “no rank” taxa without disarranging the rank hierarchy already established by the ranked taxa. In the algorithm, we also established a priority scale among the ranks (Table 1) to help in choosing a single rank to be assigned to a “no rank” node with two or more candidate ranks. This procedure favors those most frequent ranks to be selected to assign a “no rank” node (nodes of type 1). We did not note a published report that takes advantage of the “no rank” taxa to fulfill all missing ranks. However, a similar but simpler approach can be found in the function “reformat” of a tool named TaxonKit [47], which could address this problem in some bioinformatics applications.

## Conclusion

Several bioinformatics analyses and tools rely on the taxonomic information provided by NCBI Taxonomy. However, working with or querying data by taxonomic-rank is not trivial because of the absence of some ranks in the taxonomic lineages and the presence of taxa without a rank throughout the taxonomic tree. In this work, we address this issue by developing an algorithm that takes the taxonomic tree from NCBI Taxonomy and makes it hierarchically complete according to the taxonomic-ranks. The final tree was named Taxallnomy, and it has 41 hierarchical levels corresponding to the 41 taxonomic-ranks that comprise the NCBI Taxonomy. From the Taxallnomy database, the user can retrieve the complete taxonomic lineage with 41 nodes, all of them with a taxonomic-rank, to all taxa available in the NCBI Taxonomy. Taxallnomy applies to any bioinformatics analyses that depend on the information from NCBI Taxonomy.

## Data Availability

Taxallnomy is freely available at http://bioinfo.icb.ufmg.br/taxallnomy/.

## Abbreviations

INSDC: International Nucleotide Sequence Database Collaboration
txid: Taxonomic identifier
NA: Node in analysis
L1: First level
RL: Redundant level
CR: Candidate ranks
LDP: Longest downward path
